# Roles and Mechanism of miR-199a and miR-125b in Tumor Angiogenesis

**DOI:** 10.1371/journal.pone.0056647

**Published:** 2013-02-20

**Authors:** Jun He, Yi Jing, Wei Li, Xu Qian, Qing Xu, Feng-Shan Li, Ling-Zhi Liu, Bing-Hua Jiang, Yue Jiang

**Affiliations:** 1 State Key Lab of Reproductive Medicine, Department of Pathology, Cancer Center, Nanjing Medical University, Nanjing, Jiangsu, People’s Republic of China; 2 Department of Pathology, Anatomy and Cell Biology, Thomas Jefferson University, Philadelphia, Pennsylvania, United States of America; 3 Department of Pathology, Nanjing Maternal and Child Care Service Center, Nanjing, Jiangsu, People’s Republic of China; 4 Faculty of Software, and College of Life Sciences, Fujian Normal University, Fuzhou, Fujian, People’s Republic of China; H.Lee Moffitt Cancer Center & Research Institute, United States of America

## Abstract

MicroRNAs (miRNAs) have been shown to be involved in different aspects of cancer biology including tumor angiogenesis. In this study, we identified that two miRNAs, miR-199a and miR-125b were downregulated in ovarian cancer tissues and cell lines. Overexpression of miR-199a and miR-125b inhibited tumor-induced angiogenesis associated with the decrease of HIF-1α and VEGF expression in ovarian cancer cells. Moreover, the levels of miR-199a and miR-125b were negatively correlated with VEGF mRNA levels in ovarian tissues. We further showed that direct targets of miR-199a and miR-125b HER2 and HER3 were functionally relevant. Forced expression of HER2 and HER3 rescued miR-199a- and miR-125b-inhibiting angiogenesis responses and Akt/p70S6K1/HIF-1α pathway. This study provides a rationale for new therapeutic approach to suppress tumor angiogenesis using miR-199a, miR-125b, or their mimics for ovarian cancer treatment in the future.

## Introduction

Ovarian cancer is the fifth leading cause of cancer death in women and the most lethal gynecologic malignancy in the Western world [1]. In the U.S., the overall lifetime risk of developing ovarian cancer is estimated to range between 1 in 56 (1.8%) and 1 in 71 (1.4%) women [2]. Despite recent advances in chemotherapeutic treatments that have improved the initial responses, the 5-year survival rate for women with advanced-stage ovarian cancer is only about 30% after initial diagnosis [3]. Consequently, a better understanding of the mechanisms leading to the initiation and progression of ovarian cancer is required to develop new targets and therapeutic strategies.

Angiogenesis is required for the cancer development and growth. Without angiogenesis, cancer cells inside the tumor will undergo apoptosis [4]. The angiogenesis switch depends on the balance of angiogenesis activators and inhibitors. Recent studies have shown that some miRNAs are involved in the regulation of vascular development and angiogenesis [5]. The global inhibition of Dicer and Drosha, two key enzymes for miRNAs biogenesis led to impaired angiogenesis [6]. Some miRNAs such as miR-10b and miR-196b have been identified to promote angiogenesis by directly regulating bone marrow-derived endothelial progenitor cells (EPCs) [7], whereas miR-126 induces angiogenesis by increasing vascular endothelial cell growth factor (VEGF) expression [8]. Conversely, miR-221 and miR-222 inhibit angiogenesis by targeting human proto-oncogene c-Kit receptors in endothelial cells [9]. Data from miRNA microarray analysis shows that some miRNAs are aberrantly expressed in ovarian cancer [10], indicating the involvement of miRNAs in ovarian cancer development. However, the roles of miRNAs in regulating angiogenesis in ovarian cancer remain to be determined. Our preliminary data indicated that miR-199a and miR-125b may be involved in angiogenesis. In this study, we plan to investigate: 1) the expression levels of miR-199a and miR-125b in human ovarian tissues and their correlation with potent angiogenesis inducer VEGF; 2) the direct roles of miR-199a and miR-125b in affecting angiogenesis; 3) what signaling molecules and pathway(s) are involved in miR-199a- and miR-125b-inhibiting angiogenesis; and 4) which direct targets of miR-199a and miR-125b are involved in angiogenesis, and miR-199a- and miR-125b-regulated pathway(s).

## Materials and Methods

### Ethics Statement

The study protocol was approved by the Nanjing Maternal and Child Care Service Center Institutional Review Board and the informed written consents were given by all the patients. No information related to the Health Insurance Portability and Accountability Act was included in the study, which qualifies for the status of NIH Exemption # 4.

### Ovarian Cancer Tumor Tissues

The tissue samples of primary epithelial ovarian cancer and normal ovarian tissues were collected by Nanjing Maternal and Child Care Service Center, Nanjing, China. These tissues were partly snap-frozen in liquid nitrogen and stored at -80°C before the analysis, and partly fixed for pathology diagnosis. In this study, we used 33 ovarian papillary serous cystadenocarcinoma and 7 normal ovarian tissues.

### Antibodies

Antibodies against p-AKT, total AKT, and p-p70S6K1 were from Cell Signaling Technology (Beverly, MA); against p70S6K1 were from Santa Cruz Biotechnology (Santa Cruz, CA); against total HER2 and HER3 were from Upstate Biotechnology (Upstate, NY); and against HIF-1α and HIF-1β were from BD Bioscience (Franklin Lakes, NJ).

### Cell Culture and Generation of Stable Cell Lines

The human ovarian cancer cells OVCAR3 and A2780 were purchased from ATCC (Manassas, VA, US). The immortalized ovarian epithelial cells IOSE386 and IOSE397 were generated by transfecting normal ovarian surface epithelial cells with the immortalizing simian virus 40 early genes [11]. These cells were cultured in RPMI 1640 medium (Invitrogen, Carlsbad, CA) supplemented with 10% fetal bovine serum (FBS). The human umbilical vein endothelial cells (HUVEC) (ATCC, Manassas, US) were cultured in EBM-2 complete medium. Stable cell lines of A2780 cells overexpressing HER2 were generated by transfecting the pBaBe vector expressing HER2 cDNA without 3′ UTR region into 293 FT (Life technologies, Grand Island, NY, US) cells to obtain infectious virus using FuGENE6 (Roche, Indianapolis, IN ). A2780 cells were infected by the virus alone or carrying HER2 for 48 h, followed by selection in medium containing 1.0 µg/ml puromycin for 7 days. Stable cell lines of OVCAR-3 cells overexpressing HER3 was generated by transfecting the pReceiver-Lv105 vector expressing HER3 cDNA without 3′ UTR region into 293 FT cell using Trans-lentiviral package kit (Open Biosystem, Huntsville, AL). OVCAR-3 cells were infected by lentivirus carrying HER3, followed by the selection with puromycin to obtain the stable cell lines. The similar method was used to generate A2780 stable cell lines expressing HIF-1α cDNA. Human HER2 cDNA clone with 3′ UTR region was purchased from Origene (Rockville, MD, US) for transient transfection.

### miRNA Transfection

The negative control miRNA precursor, miR-199a and miR-125b precursors were obtained from Applied Biosystem (Austin, TX). Cells were cultured in 6-well plate to reach 60% confluency, and transfected using miRNA precursors at 30 nM using X-treme Gene siRNA transfection reagent (Roche, Mannheim, Germany) according to the manufacturer’s instruction. Total proteins and RNAs were prepared from the cells for analysis 60–70 h after the transfection.

### Plasmid Constructs

The 3′UTR-luciferase reporter constructs containing the 3′UTR regions of HIF-1α with wild-type and mutant binding sites of miR-199a were amplified using PCR method. Total cDNAs was obtained from A2780 cells. The PCR products were cloned into the pMiR-luc reporter vector (Ambion) between Sac I and Hind III sites, immediately downstream of the luciferase gene. The mutant 3′UTR constructs were made by introducing four mismatch mutations into the putative seed regions of HIF-1α. All the constructs containing 3′UTR inserts were sequenced and verified.

### RT-PCR and Taqman qRT-PCR

Total RNAs were extracted using Trizol (Life technologies, Carlsbad, CA). The cDNA synthesis was performed using oligo(dT)_18_ primers and M-MLV reverse transcriptase. For VEGF mRNA analysis, the primers used for PCR were as follows: VEGF forward primer, 5′-TCGGGCCTCCGAAACCATGA-3′; VEGF reverse primer, 5′-CCTGGTGAGAGATCTGGTTC-3′; GAPDH forward primer, 5′-CCACCCATGGCAAATTCCATGGCA-3′; and GAPDH reverse primer, 5′-TCTAGACGGCAGGTCAGGTCCACC-3′. The PCR amplification was performed at: 95°C for 5 min, followed by 28 cycles of 95°C for 1 min, 59°C for 45 s, 72°C for 1 min. Stem-loop RT-PCR was used to determine miRNA expression levels in cells and tumor tissues. The PCR primer pairs for miR-125b, miR-199a and U6 were synthesized by Shanghai Sangon Biological Engineering Technology and Services (Shanghai, China). The primers for miR-125b, miR-199a and U6 were as follows: miR-125b were 5′-GCAACCTTGCGACTATAACCATCACAAGTTA-3′ (stem-loop), 5′-GGCAACCTTGCGACTATAACCA-3′ (sense), 5′-GTTTCCTCTCCCTGAGACCCTA-3′ (antisense). miR-199a were 5′-AAGGCGATTGATACGAGTCAGAACAGGTA-3′ (stem-loop), 5′-AGAAGGCGATTGATACGAGTCA-3′ (sense), 5′-GGTCTCCCCAGTGTTCAGATA-3′ (antisense). U6 were 5′-GTCGTATCCAGTGCAGGGTCCGAGGTATTCGCACTGGATACGACAAAATATG-3′ (stem-loop), 5′-GTGCAGGGTCCGAGGT-3′ (sense), 5′-CGCTTCGGCAGCACAT-3′ (antisense). The expression levels of miR-125b, miR-199a in samples were measured in terms of threshold cycle value and normalized to U6 levels as an internal control. All reactions were performed in triplicates.

For Taqman RT-PCR, small RNAs were extracted from cultured cells using the mirVana miRNA isolation kit (Ambion, Austin, TX). Two-step Taqman real-time PCR analysis was performed to assess miRNA levels using Taqman miRNA reverse transcription kit and Taqman universal PCR master mix (Applied Biosystem, Austin, TX, USA) in accordance with manufacturer’s instructions. Normalization was performed with U6 RNA. Each analysis was performed in three replicates and three technical replicates within each experiment.

### Real-time RT-PCR for VEGF

Total RNAs in tissues were extracted using Trizol. Reverse transcription reactions were performed by using High Capacity RNA-to cDNA according to the manufacturer’s instructions. The 100 ng of RT product was used for PCR reaction using Power SYBR Green PCR Master Mix Kit (Applied Biosystems, Carlsbad, CA, USA). The primers of VEGF are: forward primer, 5′-CGAGGGCCTGGAGTGTG-3′; reverse primer, 5′-CCGCATAATCT GCATGGTGAT-3′. The primers of GAPDH are: forward primer, 5′-ATGGGTGTGAACCATGA GAAGTATG-3′; reverse primer: 5′GGTGCAGGAGGCATTGCT-3′.

### Luciferase Reporter Assay

Cells were seeded on 12-well plates and cultured to 50–60% confluency. The cells were transiently transfected with HIF-1α 3′UTR miRNA luciferase (luc) reporters or VEGF luc reporter (0.4 µg), β-gal plasmid (0.2 µg), pre-miRNA precursor (0.4 µg) using lipofectamine according to the manufacturer’s instruction. The cells were harvested and lysed with reporter lysis buffer (Promega, Madison, WI) 48 h after the transfection. The luc activities in the cellular extracts were determined using the luc assay system (Promega). The β-gal activity was measured with assay buffer (200 mM phosphate, 2 mM MgCl2, 100 mM β-mercaptoethanol, 1.33 mg/ml o-nitrophenyl β-D-galactopyranoside). The relative luc activity was calculated by the ratio of luc/β-gal activity, and normalized to that of the control cells.

### Western Blotting

The cells were harvested and washed in cold 1× PBS buffer, and lysed in ice-cold RIPA buffer supplemented with protease inhibitors on ice for 30 min. Cell debris was discarded by centrifugation at 12,000 rpm for 10 min at 4°C. The protein concentrations were assayed using Bio-Rad protein assay reagents. Protein samples were subjected to immunoblotting analysis.

### Tube Formation Assay

Human umbilical vein endothelial cells (HUVEC) were cultured in EBM-2 complete medium, and switched to EBM-2 basal medium containing 0.2% FBS for 24 hours to be used for the tube formation assay. The conditioned media were prepared from different ovarian cancer cells by replacing normal culture medium with serum-reduced medium (1% FBS). After culture for 24 h, the serum-reduced medium was collected and stored at −20°C for later use. The HUVEC cells were trypsinized, counted and resuspended in EBM-2 basic medium. Then the cells were mixed with equal volume of the conditioned medium and seeded on Matrigel-pretreated 96-well plate at density of 2×10^4^ cells/well. Tube formation was observed under light microscope after 12 h, and photographed. The total length of the tubes for each well was measured using CellSens Standard software. Each treatment has six replicates.

### CAM Assay

White Leghorn fertilized chicken eggs were incubated at 37°C under constant humidity. Different ovarian cancer cells and control cells were transfected with certain miRNA precursors or treated as specifically indicated. These cells were trypsinized, counted and resuspended in the serum-free medium. The cell suspensions were mixed with Matrigel at 1∶1 ratio and implanted onto the chorioallantoic membranes (CAM) of chicken eggs at Day 9. Tumor angiogenesis responses were analyzed 5 days after the implantation. The tumor/Matrigel plugs were trimmed off CAM and photographed. The number of blood vessels as the index of angiogenesis was obtained by counting the branches of blood vessels in three representative areas (1.5 mm^2^) by two observers in a double blind manner.

### Statistic Analysis

All the results were obtained from at least three independent experiments. Most results were presented as mean ± SE from independent experiments, and analyzed by Student’s *t* test, One-way ANOVA, and/or Pearson correlation analysis. All results were analyzed by SPSS for Windows, version 11.5. Differences were considered significant at a value of *P*≤0.05.

## Results

### MiR-199a and miR-125b are Down-regulated in Ovarian Cancer Tissues

**Figure 1:**
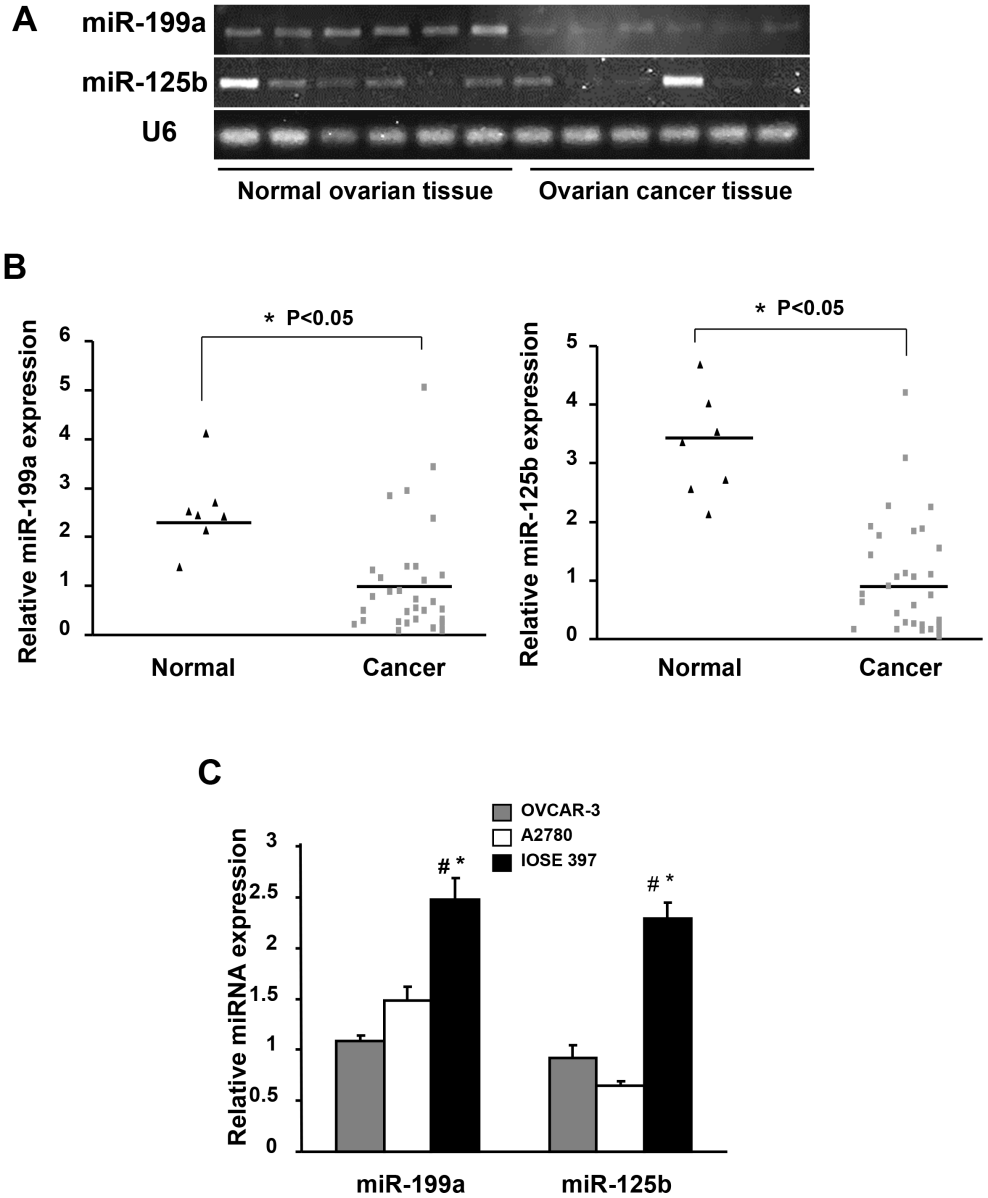
MiR-199a and miR-125b are down-regulated in ovarian cancer tissues and cells. (A) The expression levels of miR-199a and miR-125b in human epithelial ovarian carcinoma tissues and normal ovarian tissues determined by RT-PCR. Total of 33 ovarian epithelial cancer tissues and 7 normal ovary tissues were analyzed. Representative samples (n = 6 for each group) are shown. The expression level of U6 was used as loading control. (B) Taqman RT-PCR was performed to assess miR-125b and miR-199a expression levels using 33 ovarian cancer tissues and 7 normal tissues. The bar refers to the mean for each group. The values were normalized to the mean of cancer tissues. *Significantly different compared with that of control (P<0.05). (C) The miR-199a and miR-125b expression levels were analyzed using Taqman qRT-PCR in ovarian cancer cell lines. Relative expression levels were represented as RQ using of 2^−ΔΔCT^ methods. Each data sample was normalized to the U6 expression level and that in OVCAR-3 cells. Mean ± SE values were from three separate experiments. ^#^Significantly different compared with OVCAR-3 (P<0.05). *Significantly different compared with A2780 (P<0.05).

According to miRNA array analysis, miR-199a and miR-125b were down-regulated in human epithelial ovarian carcinoma tissues compared to normal ovarian tissues [10], [12]. We used both regular RT-PCR and Taqman qRT-PCR to examine miR-199a and miR-125b expression levels in 33 human ovarian serous adenocarcinomas tissues and 7 normal human ovarian tissues, and found that miR-199a and miR-125b expression levels were significantly lower in ovarian cancer tissues than those in normal tissues (Figures 1A, B). Similarly miR-199a and miR-125b expression levels in ovarian cancer cell lines OVCAR-3, A2780 were much lower than those in immortalized ovarian epithelial cell line IOSE397 (Figure 1C).

### MiR-199a and miR-125b Suppress Tumor Angiogenesis via HIF-1α/VEGF Pathway

**Figure 2:**
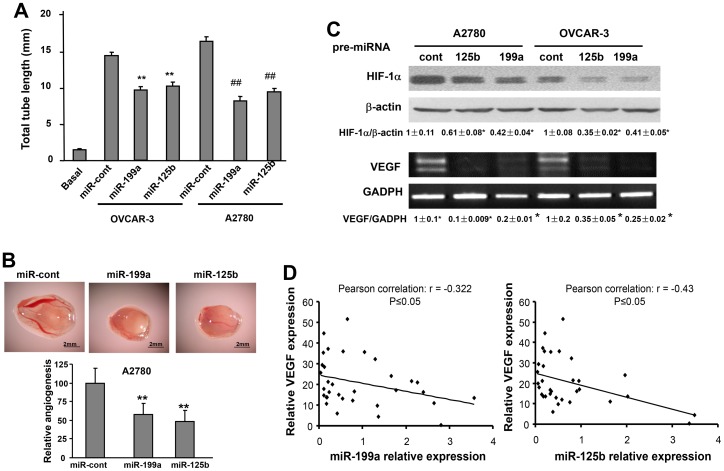
MiR-199a and miR-125b suppress tumor angiogenesis associated with reduction of HIF-1α and VEGF expression. (A) HUVEC cells were cultured in serum free medium overnight and re-suspended in basic EBM-2 medium. To perform the tube formation assay, HUVEC cells were incubated in basic EBM-2 medium; conditioned medium prepared from OVCAR-3 or A2780 transfected with pre-miR-control, pre-miR-125b and pre-miR-199a, respectively. Tube formation was determined under light microscope in 12 h. Total tube length (mm) was presented as mean ± SE from six replicates for each treatment. **Significantly different compared with OVCAR-3 control (P<0.01). ^##^Significantly different compared with A2780 control (P<0.01). (B) A2780 cells were transfected with 30 nM pre-miR-125b, pre-miR-199a and pre-miR-control precursor, respectively. After transfection (24 h), 2×10^6^ cells were trypsinized, suspended, and mixed with equal volume of Matrigel, and implanted onto the chicken CAMs of 10-day-old chicken embryos. The branches of blood vessels were counted as the index of angiogenesis was obtained from the CAMs of 8–10 embryos per treatment 96 h after implantation. The data represent as mean ± SE of blood vessel numbers, which were normalized to that of the control. **Significantly different compared with that of the control (P<0.05). (C) A2780 cells and OVCAR-3 cells were cultured under normoxic condition, and transiently transfected as above to analyze HIF-1α and β-actin protein expression by immunoblotting and VEGF mRNA levels (VEGF-165 and VEGF-121 isoforms) by RT-PCR. Quantification was performed by scanning densitometry. The results are obtained from triplicate experiments and presented as mean ± SE. *Significantly difference compared with the same cell lines transfected with miR-cont. (D) VEGF mRNA levels in human ovarian cancer tissues (n = 33) were analyzed by SYBR Green qRT-PCR. The Pearson correlations were analyzed between miR-125b or miR-199a expression and its corresponding VEGF expression in cancer tissues.

Angiogenesis is the essential process for tumor development [4]. We evaluated the potential roles of miR-199a and miR-125b in angiogenesis. First, we performed tube formation assay using human umbilical vein endothelial cells (HUVEC). No tube formation by HUVEC cells was observed using EBM-2 basic medium, whereas tube formation was strongly induced using conditioned medium prepared from ovarian cancer cells OVCAR-3 or A2780. The conditioned medium from OVCAR-3 and A2780 cells overexpressing miR-199a or miR-125b induced significantly less tube formation than that from the cells expressing negative control miRNA (Figure 2A). Next, we employed chicken chorioallantoic membrane (CAM) assay for studying angiogenesis *in vivo*. MiR-199a and miR-125b overexpression in ovarian cancer cells decreased their ability of inducing angiogenesis by 40% and 50%, respectively (Figure 2B). VEGF is the major angiogenesis inducer both in physiological and pathological processes, while HIF-1α is the major regulator of VEGF transcriptional activation through the binding to the hypoxia response element of VEGF promoter [13]. To examine whether HIF-1α/VEGF pathway is involved in this process, the expression levels of HIF-1α protein and VEGF mRNA were determined. As expected, miR-199a and miR-125b remarkably inhibited HIF-1α and VEGF expression in both cell lines (Figure 2C). This result suggests that miR-199a and miR-125b repress angiogenesis potential of cancer cells through inhibiting HIF-1α/VEGF expression. To test whether these findings have potential clinical application, we measured VEGF mRNA levels in ovarian cancer tissues by real time RT-PCR and performed the correlation analysis of VEGF levels and relative expression levels of miR-199a or miR-125b in these tissue samples. An inverse correlation was observed between VEGF levels, and miR-199a or miR-125b expression (P≤0.05) (Fig. 2D), suggesting that the expression of miR-199a or miR-125b regulates VEGF in human cancer tissues. It has been reported that HIF-1α is the direct target of miR-199a in cardiac myocytes [14], and this prompted us to construct HIF-1α 3′UTR reporter for validation. However, we were unable to confirm the direct interaction between miR-199a and HIF-1α in ovarian cancer cells (Figure S1), suggesting an indirect effect of miR-199a on HIF-1α expression.

### HER2 and HER3 Proteins are Highly Expressed in Ovarian Cancer

**Figure 3:**
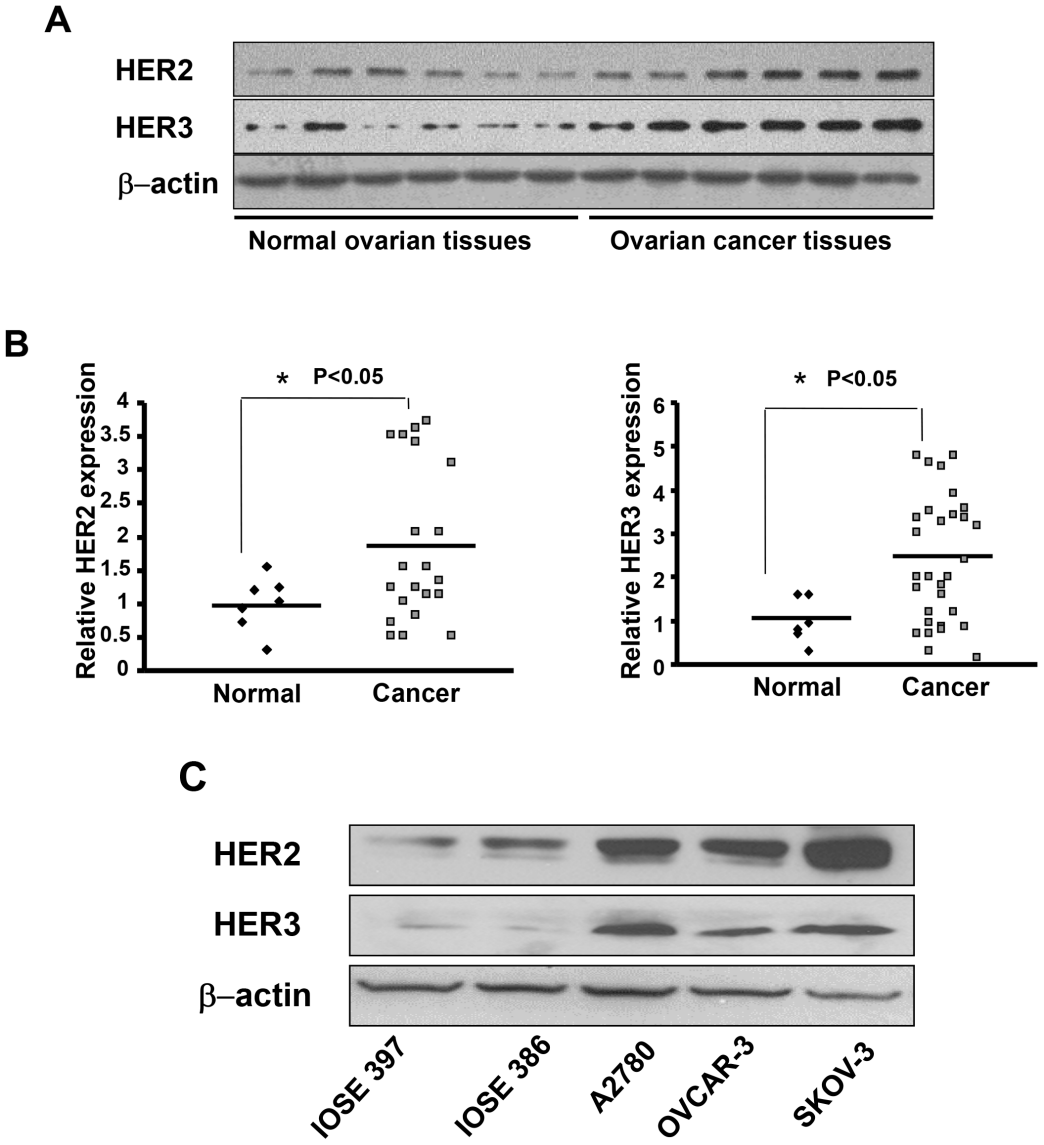
HER2 and HER3 protein expression levels in ovarian cancer tissues. (A) The protein levels of HER2 and HER3 were evaluated in human ovarian cancer tissues and normal ovarian tissues by immunoblotting (6 representatives HER2 or HER3 positive samples are shown for both groups). The level of β-actin was used as loading control. (B) Scanning densitometry was used to quantify the relative expression levels of HER2 or HER3. The scattered graphs showed HER2 or HER3 expression levels in HER2 or HER3 positive samples in normal tissues and cancer tissues. The values were normalized to the average value of normal tissues. (C) Expression levels of HER2 and HER3 in ovarian cancer cell lines SKOV3, A2780, OVCAR-3, and immortalized ovarian epithelial cell lines IOSE397, IOSE396 were analyzed by immunoblotting.

Recent findings from our group and other group show that HER2 is a direct target of miR-199a and HER3 is a direct target of both miR-199a and miR-125b in A2780 and OVCAR-3 cells [15], [16]. Given the important roles of HER2 and HER3, there are great interests in investigating HER2 and HER3 status in human cancers; however, there is notable variability across the studies. In this study we analyzed 33 human ovarian serous adenocarcinomas and 7 normal ovarian tissues. Overall, 21 out of 33 cancer samples and all 7 normal ovarian samples are HER2 positive whereas 29 out of 33 cancer samples and 6 out of 7 normal samples are HER3 positive. Scanning densitometry reveals that the average expression of HER2 in 21 HER2 positive ovarian cancer tissues was higher relative to 7 HER2 positive normal ovarian tissues (Figure 3A, B). The same trend was observed for HER3 expressions. To test HER2 and HER3 expression levels in ovarian cancer cells, we found that SKOV3, OVCAR-3 and A2780 cells have much higher expression levels than immortalized ovarian epithelial cell lines IOSE 386 and IOSE 397 (Figure 3C). Taken together, these results show that HER2 and HER3 proteins are generally increased in ovarian cancer tissues and cancer cell lines.

### MiR-199a and miR-125b Inhibit Angiogenesis via HER2 and HER3

**Figure 4:**
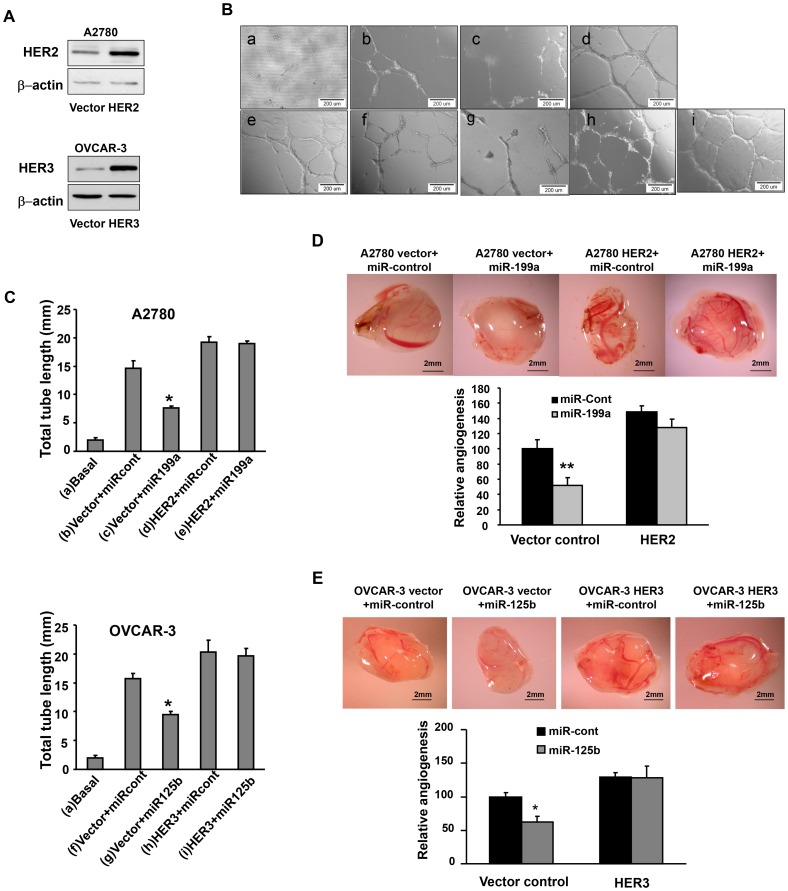
Overexpression of HER2 and HER3 reverses miR-199a- and miR-125b-inhibited tumor angiogenesis. (A) Immunoblotting to confirm establishment of an A2780 cell line stably overexpressing HRE2 and OVCAR-3 cell line stably overexpressing HER3 using pBABEpuro and pReceiver-Lv105 vector, respectively. (B) Tube formation assay using HUVEC cells was described as in Figure 2. (C) Total tube lengths for each treatment were analyzed and presented as mean ± SE (millimeter) from six replicates for each treatment. *Significantly different vs vector+miR-control (P<0.05). (D, E) A2780 and OVCAR cells were transfected pre-miR-control, pre-miR-199a or pre-miR-125b, respectively; and implanted onto the CAMs to perform angiogenesis assay as in Figure 2. The representative images from each group were shown here. The total number of blood vessels in each group was quantified. *,**Significantly different compared with that of the same cell line transfected with pre-miR-control with *P<0.05 and **P<0.01.

To determine whether miR-199a and miR-125b decrease angiogenesis potential by targeting HER2 and HER3, we established ovarian cancer stable cell lines overexpressing HER2 and HER3 using HER2 and HER3 cDNA plasmids without 3′ UTR regions, respectively. The HER2 and HER3 protein expression was confirmed in these stable cell lines by immunoblotting (Figure 4A). Tube formation assay was initially conducted to evaluate angiogenesis effects (Figures 4B, C). No tube formation was observed by HUVEC cells cultured in basic medium, while tube formation was induced by HUVEC cells cultured in conditioned medium prepared from A2780 or OVCAR-3 cells. The ectopic expression of miR-199a or miR-125b in OVCAR-3 and A2780 cells significantly impaired the tube formation. The forced expression of HER2 and HER3 in the cells completely restored the tube formation abilities even in the cells transfected with miR-199a or miR-125b precursor, indicating that lack of HER2 and HER3 3′UTR regions makes cells resistant to the miRNAs regulation. To further examine the roles of HER2 and HER3 in tumor-induced angiogenesis, we analyzed tumor angiogenesis responses *in vivo* by CAM model. The results were entirely consistent with the data from the tube formation assay. Tumor-induced angiogenesis was impaired by ectopic expression of miR-199a or miR-125b and was reversed by HER2 or HER3 overexpression (Figures 4D, E). To further confirm the negative regulation of miR-199a/125b on HER2 and/or HER3, OVCAR-3 cells were transiently transfected with HER cDNA containing miR-199a binding sites and miR-cont or miR-199a precursor to get conditioned medium. Tube formation assay was performed to assess the angiogenic potential of cancer cells. The results showed that miR-199a was able to decrease the tube formation (Figure. S2). Collectively, these results suggest that miR-199a and miR-125b inhibit angiogenesis by directly targeting and inhibiting HER2 and HER3 expression.

### Overexpression of HER2 or HER3 in Ovarian Cancer Cells Reversed the miRNA Effects via Akt/p70S6K1/HIF-1α/VEGF Pathway

**Figure 5:**
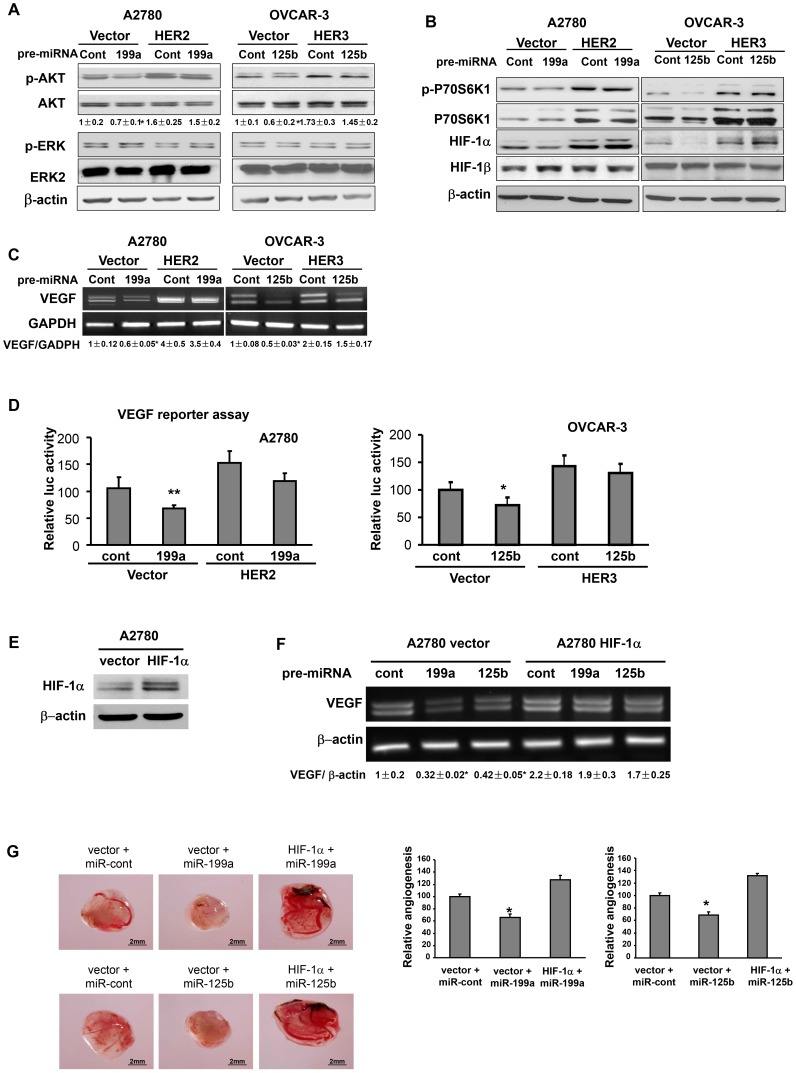
Overexpression of HER2 or HER3 in ovarian cancer cells reversed miR-199a and miR-125b inhibitory effects on Akt/p70S6K1/HIF-1α/VEGF pathway. (A, B) A2780 vector, A2780 HER2, OVCAR-3 vector and OVCAR-3 HER3 cells were cultured under normoxic condition, and were transfected with pre-miR control, pre-miR-199a and pre-miR-125b precursor at the final concentration of 30 nM. The cells were harvested and analyzed 70 h after transfection for protein levels of p-Akt, total Akt, p-p70S6K1, p70S6K1, HIF-1α and HIF-1β. (C) RT-PCR was used to assess the VEGF mRNA levels in stable cells transfected with miR-control, miR-199a or miR-125b. (D) Cells were seeded in 12-well plates and transiently co-transfected with human VEGF luciferase reporter, pre-miRNAs and β-galactosidase plasmids. The cells were cultured for 48 h after transfection and relative luciferase activities were measured as described in Method section. (E) A2780 cells stably overexpressing HIF-1α was established by infecting A2780 cells with retrovirus carrying HIF-1α. (F) A2780 cells and A2780/HIF-1α cells were transiently transfected with the indicated miRNAs, and harvested 70 h later to analyze VEGF mRNA levels by RT-PCR as indicated in the figure. (G) A2780 vector control cells and A2780 HIF-1α cells were transfected with pre-miR-control, pre-miR-199a or pre-miR-125b, respectively; then 2×10^6^ cells were used to perform angiogenesis assay as described in Figure 2. *Significantly different compared with that of the vector cell line transfected with pre-miR-control (P<0.05).

AKT and ERK1/2 are known downstream mediators of HER2 and HER3 [17]. The aberrant activation of AKT or ERK is considered to be hallmarks of cancer [18]. To study whether miR-199a and miR-125b utilize these two pathways, ovarian cancer cells expressing vector alone, HER2, or HER3 were transfected with miR-199a and miR-125b precursor; and analyzed for AKT and ERK expression levels. Overexpression of miR-199a and miR-125b resulted in decreased p-AKT levels, but not p-MAPK level (Figure 5A). Forced expression of HER2 or HER3 alone was sufficient to restore p-AKT expression levels in the cells with miR-199a and miR-125b overexpression. P70S6K1 is a downstream target of AKT and mTOR [19]. P70S6K1 may regulate HIF-1α expression and tumor angiogenesis [20]. Overexpression of HER2 in breast cancer cells increased VEGF protein synthesis via p70S6K1, resulting in enhanced angiogenesis [21]. In this study, we found that miR-199a or miR-125b inhibited phosphorylation of p70S6K1 and downstream HIF-1α and VEGF expression, while enforced expression of HER2 or HER3 reversed such inhibitory effects (Figures 5B, C). No significant difference was observed in HER2 or HER3 overexpressing cells when transfected with miR-199a or miR-125b. Similar results were obtained from VEGF luciferase reporter activity assay (Figure 5D).

HIF-1 is known to regulate VEGF expression at the transcriptional activation level [13]. We also examined the role of HIF-1α in miR-199a- and miR-125b-inhibited angiogenesis by establishing an A2780 cell stably overexpressing HIF-1α (Figure 5E). As we expected, VEGF expression was attenuated by miR-199a and miR-125b overexpression, and rescued by HIF-1α forced expression (Figure 5F). CAM assay was performed and showed that miR-199a or miR-125b alone was sufficient to inhibit A2780 cell-induced angiogenesis, whereas HIF-1α overexpression restored miR-199a- and miR-125b-inhibited angiogenesis (Figure 5G), suggesting that inhibitory effects of miR-199a- and miR-125b on tumor-induced angiogenesis are mediated by HIF-1α downregulation.

## Discussion

A number of recent studies have identified altered miRNA signatures in ovarian tumors. It has been reported that miR-200a, miR-141, miR-200c and miR-200b were up-regulated whereas miR-199a, miR-140, miR-145 and miR-125b were down-regulated in ovarian tumor tissues [22]. Another report showed high expression of miR-200, miR-141, miR-18a, miR-93, miR-429 and low expression of let-7b and miR-199a were associated with poor prognosis in serous ovarian carcinoma [10]. In this study, we utilized human ovarian epithelial cancer tissues and ovarian cancer cell lines A2780 and OVCAR-3 to analyze miR-199a and miR-125b expression levels, and showed that both of them are down-regulated in ovarian cancer tissues and cells. The functional analysis revealed that ectopic expression of miR-199a or miR-125b suppresses tumor angiogenesis both *in vitro* and *in vivo*, indicating that these two miRNAs have anti-angiogenic properties.

VEGF is a key proangiogenic activator which can be activated at transcriptional level by HIF-1 [13]. To understand the potential mechanism of miR-199a/125b inhibiting angiogenesis, we initially analyzed the effect of miR-199a/125b on HIF-1/VEGF expression. Overexpression of miR-199a/125b dramatically downregulates HIF-1α protein expression as well as VEGF mRNA levels, suggesting that miR-199a/125b suppresses tumor angiogenesis by decreasing the expression of HIF-1α and VEGF.

HER2/3 is known to be involved in regulation of HIF-1 activity and stability [23]. To identify potential upstream molecules of HIF-1, we investigated the roles of HER2 and HER3 in miR-199a/125b inhibitory effect on angiogenesis. HER2 and HER3 are overexpressed in ovarian cancer tissues (Figure 3). Our unpublished data and other researchers showed that miR-125b targets HER2 and/or HER3 in the cells [16]. Overexpression of HER2 or HER3 is sufficient to restore inhibitory effects of miR-199a and miR-125b on tumor angiogenesis, suggesting that HER2 and HER3 are functional targets of miR-199a and miR-125b in regulating angiogenesis.

We further explored the pathways that may mediate miR-199a and miR-125b inhibited HIF-1/VEGF expression. HER2/3 signaling is linked to PI3K/Akt pathways [24]. We demonstrate that delivery of exogenous miR-199a and miR-125b deactivated PI3K/Akt pathway. One of Akt downstream effectors is p70S6 kinase, a serine-threonine kinase that regulates protein translation by directly phosphorylating ribosomal protein S6 [25]. In this study, overexpression miR-199a or miR-125b was sufficient to inhibit p70S6K1 activation, suggesting HERs/Akt/p70S6K1/HIF-1α pathway is involved in transmitting the biological effects of miR-199a or miR-125b. It was reported that HER2 overexpression enhanced VEGF mRNA level through two promoter elements [26]. One is the hypoxia responsive element regulated by HIF-1, and the other is the core promoter region that is controlled through two adjacent SP1 binding sites. Here we demonstrate that miR-199a/125b inhibit VEGF expression levels through transcriptional activation via HIF-1α expression. It was reported that HIF-1α was targeted by miR-199a in myocytes [14]. But we found that miR-199a inhibited HIF-1α protein expression through HER2 and HER3 instead of directly targeting HIF-1α in ovarian cancer cells (Figure S1). Our current study demonstrates that ectopic expression of miR-199a or miR-125b is sufficient to inhibit tumor angiogenesis; identifies the functional targets and pathways involved in miR-199a- and miR-125b-inhibited tumor angiogenesis; and provides a rationale for new therapeutic approach to suppress tumor-associated angiogenesis using miR-199a or miR-125b mimics for ovarian cancer treatment in the future.

## Supporting Information

Figure S1 **HIF-1α is not the direct target of miR-199a in ovarian cancer cells.** Luciferase reporter constructs containing HIF-1α wild-type and mutant 3′UTR regions were constructed as described in Material and Methods. Each luciferase construct was co-transfected with miRNA precursors and β-gal plasmid into the cells. The luciferase activities were presented as relative luciferase activity normalized to those of HIF-1α wild-type 3′UTR reporter and negative control miRNA precursor (pre-miR-control). The results are obtained from triplicate experiments and presented as mean ± SE.
(TIF)

Figure S2 **miR-199a inhibits tube formation in cells by overexpressing HER2 cDNA with 3′ UTR region containing miR-199a binding site.** A) OVCAR-3 cells were infected with lentivirus carrying HER2 cDNA with 3′ UTR region, then transiently cotransfected with miR-control or miR-199a precursor as indicated. HER2 protein levels were determined 72 h after transfection by immunoblotting. B) HUVEC cells were cultured in serum free medium overnight and re-suspended in basic EBM-2 medium. The conditioned medium was prepared from OVCAR-3 cells as treated above. Tube formation assay was performed as described in Material and Methods. Upper: Representative images were shown. Scale bar: 200 µm. Lower: Total tube lengths (mm) were presented as mean ± SE from six replicates for each treatment. *Significantly different compared with control. ^#^Significantly different compared with HER2/miR-cont.
(TIF)

## References

[1] Jemal A, Siegel R, Ward E, Hao Y, Xu J, et al (2008) Cancer statistics, 2008. CA Cancer J Clin 58: 71–96. 18287387 10.3322/CA.2007.0010

[2] Jemal A, Siegel R, Ward E, Murray T, Xu J, et al (2007) Cancer statistics, 2007. CA Cancer J Clin 57: 43–66. 17237035 10.3322/canjclin.57.1.43

[3] Edwards BK, Brown ML, Wingo PA, Howe HL, Ward E, et al. 2005) Annual report to the nation on the status of cancer, 1975–2002, featuring population-based trends in cancer treatment. J Natl Cancer Inst 97: 1407–1427. 16204691 10.1093/jnci/dji289

[4] Folkman J (1971) Tumor angiogenesis: therapeutic implications. N Engl J Med 285: 1182–1186. 4938153 10.1056/NEJM197111182852108

[5] Weis SM, Cheresh DA (2011) Tumor angiogenesis: molecular pathways and therapeutic targets. Nat Med 17: 1359–1370. 22064426 10.1038/nm.2537

[6] Kuehbacher A, Urbich C, Zeiher AM, Dimmeler S (2007) Role of Dicer and Drosha for endothelial microRNA expression and angiogenesis. Circ Res 101: 59–68. CIRCRESAHA.107.153916 [pii];10.1161/CIRCRESAHA.107.153916 [doi]. 10.1161/CIRCRESAHA.107.153916 17540974

[7] Plummer PN, Freeman R, Taft R, Vider J, Sax M, et al.. (2012) MicroRNAs regulate tumor angiogenesis modulated by endothelial progenitor cells. Cancer Res. 0008–5472.CAN-12–0271 [pii];10.1158/0008-5472.CAN-12-0271 [doi]. 10.1158/0008-5472.CAN-12-0271 22836757

[8] Sasahira T, Kurihara M, Bhawal UK, Ueda N, Shimomoto T, et al.. (2012) Downregulation of miR-126 induces angiogenesis and lymphangiogenesis by activation of VEGF-A in oral cancer. Br J Cancer 107: 700–706. bjc2012330 [pii];10.1038/bjc.2012.330 [doi]. 10.1038/bjc.2012.330 PMC3419968 22836510

[9] Staszel T, Zapala B, Polus A, Sadakierska-Chudy A, Kiec-Wilk B, et al.. (2011) Role of microRNAs in endothelial cell pathophysiology. Pol Arch Med Wewn 121: 361–366. AOP18 [pii]. 21946298

[10] Nam EJ, Yoon H, Kim SW, Kim H, Kim YT, et al (2008) MicroRNA expression profiles in serous ovarian carcinoma. Clin Cancer Res 14: 2690–2695. 18451233 10.1158/1078-0432.CCR-07-1731

[11] Maines-Bandiera SL, Kruk PA, Auersperg N (1992) Simian virus 40-transformed human ovarian surface epithelial cells escape normal growth controls but retain morphogenetic responses to extracellular matrix. Am J Obstet Gynecol 167: 729–735. 1326894 10.1016/s0002-9378(11)91579-8

[12] Yang H, Kong W, He L, Zhao JJ, O’Donnell JD, et al (2008) MicroRNA expression profiling in human ovarian cancer: miR-214 induces cell survival and cisplatin resistance by targeting PTEN. Cancer Res 68: 425–433. 18199536 10.1158/0008-5472.CAN-07-2488

[13] Forsythe JA, Jiang BH, Iyer NV, Agani F, Leung SW, et al (1996) Activation of vascular endothelial growth factor gene transcription by hypoxia-inducible factor 1. Mol Cell Biol 16: 4604–4613. 8756616 10.1128/mcb.16.9.4604 PMC231459

[14] Rane S, He M, Sayed D, Vashistha H, Malhotra A, et al (2009) Downregulation of miR-199a derepresses hypoxia-inducible factor-1alpha and Sirtuin 1 and recapitulates hypoxia preconditioning in cardiac myocytes. Circ Res 104: 879–886. 19265035 10.1161/CIRCRESAHA.108.193102 PMC3332328

[15] He J, Xu Q, Jing Y, Agani F, Qian X, et al (2012) Reactive oxygen species regulate ERBB2 and ERBB3 expression via miR-199a/125b and DNA methylation. EMBO Rep 13: 1116–1122. 23146892 10.1038/embor.2012.162 PMC3512405

[16] Scott GK, Goga A, Bhaumik D, Berger CE, Sullivan CS, et al (2007) Coordinate suppression of ERBB2 and ERBB3 by enforced expression of micro-RNA miR-125a or miR-125b. J Biol Chem 282: 1479–1486. 17110380 10.1074/jbc.M609383200

[17] Engelman JA, Cantley LC (2006) The role of the ErbB family members in non-small cell lung cancers sensitive to epidermal growth factor receptor kinase inhibitors. Clin Cancer Res 12: 4372s–4376s. 16857813 10.1158/1078-0432.CCR-06-0795

[18] Testa JR, Tsichlis PN (2005) AKT signaling in normal and malignant cells. Oncogene 24: 7391–7393. 16288285 10.1038/sj.onc.1209100

[19] Abe Y, Yoon SO, Kubota K, Mendoza MC, Gygi SP, et al (2009) p90 ribosomal S6 kinase and p70 ribosomal S6 kinase link phosphorylation of the eukaryotic chaperonin containing TCP-1 to growth factor, insulin, and nutrient signaling. J Biol Chem 284: 14939–14948. 19332537 10.1074/jbc.M900097200 PMC2685676

[20] Liu LZ, Zheng JZ, Wang XR, Jiang BH (2008) Endothelial p70 S6 kinase 1 in regulating tumor angiogenesis. Cancer Res 68: 8183–8188. 18829578 10.1158/0008-5472.CAN-08-0819

[21] Klos KS, Wyszomierski SL, Sun M, Tan M, Zhou X, et al (2006) ErbB2 increases vascular endothelial growth factor protein synthesis via activation of mammalian target of rapamycin/p70S6K leading to increased angiogenesis and spontaneous metastasis of human breast cancer cells. Cancer Res 66: 2028–2037. 16489002 10.1158/0008-5472.CAN-04-4559

[22] Iorio MV, Visone R, Di Leva G, Donati V, Petrocca F, et al (2007) MicroRNA signatures in human ovarian cancer. Cancer Res 67: 8699–8707. 17875710 10.1158/0008-5472.CAN-07-1936

[23] Laughner E, Taghavi P, Chiles K, Mahon PC, Semenza GL (2001) HER2 (neu) signaling increases the rate of hypoxia-inducible factor 1alpha (HIF-1alpha) synthesis: novel mechanism for HIF-1-mediated vascular endothelial growth factor expression. Mol Cell Biol 21: 3995–4004. 11359907 10.1128/MCB.21.12.3995-4004.2001 PMC87062

[24] Scaltriti M, Baselga J (2006) The epidermal growth factor receptor pathway: a model for targeted therapy. Clin Cancer Res 12: 5268–5272. 17000658 10.1158/1078-0432.CCR-05-1554

[25] Dufner A, Thomas G (1999) Ribosomal S6 kinase signaling and the control of translation. Exp Cell Res 253: 100–109. 10579915 10.1006/excr.1999.4683

[26] Pore N, Liu S, Shu HK, Li B, Haas-Kogan D, et al (2004) Sp1 is involved in Akt-mediated induction of VEGF expression through an HIF-1-independent mechanism. Mol Biol Cell 15: 4841–4853. 15342781 10.1091/mbc.E04-05-0374 PMC524732

