# Supplementary figures and images for: Roles and Mechanism of miR-199a and miR-125b in Tumor Angiogenesis
Source: PLoS One. 2013 Feb 20;8(2):e56647. doi: 10.1371/journal.pone.0056647 (PMC3577861; doi:10.1371/journal.pone.0056647)

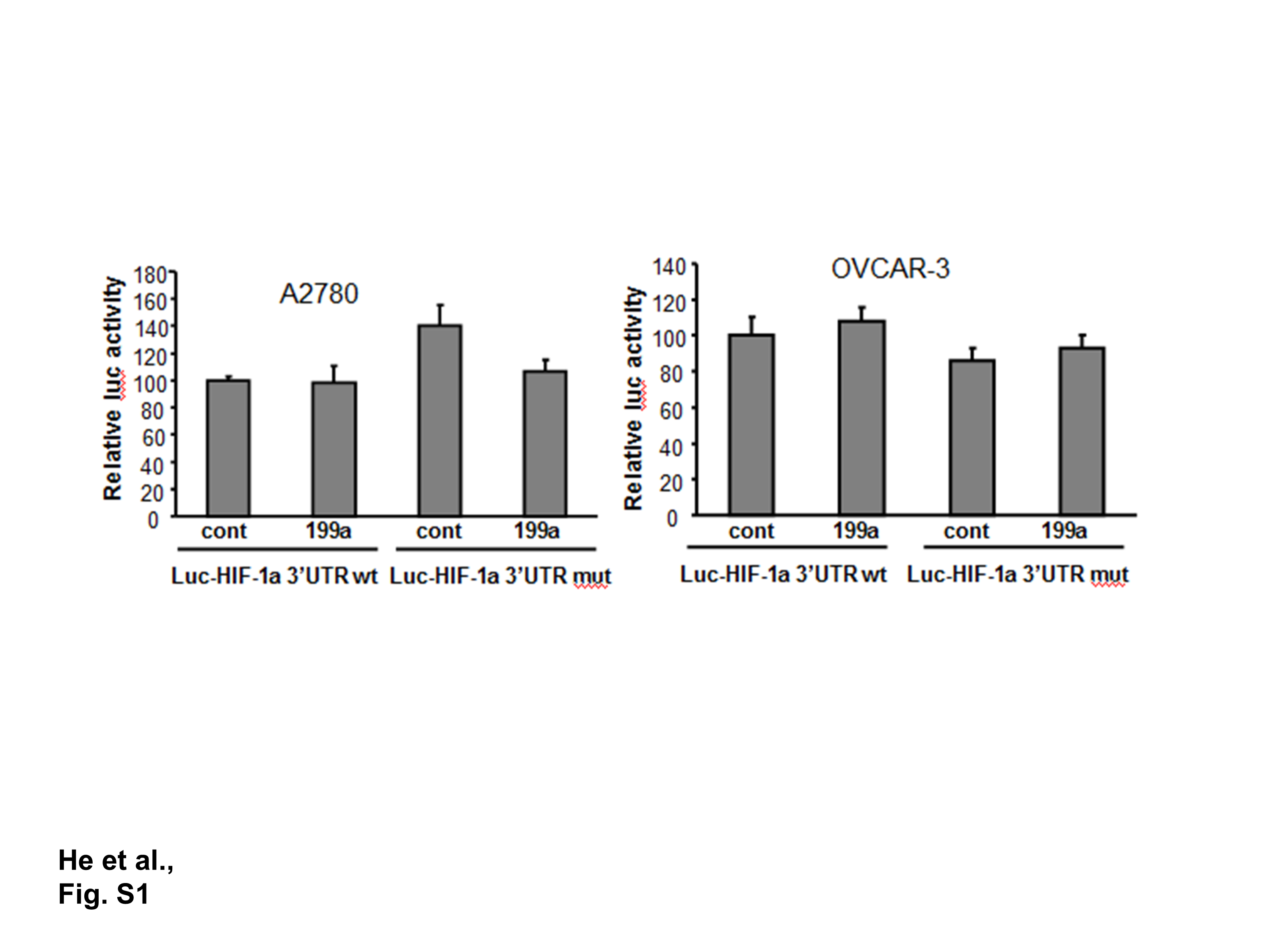

Supplement: Figure S1 — HIF-1α is not the direct target of miR-199a in ovarian cancer cells. Luciferase reporter constructs containing HIF-1α wild-type and mutant 3′UTR regions were constructed as described in Material and Methods. Each luciferase construct was co-transfected with miRNA precursors and β-gal plasmid into the cells. The luciferase activities were presented as relative luciferase activity normalized to those of HIF-1α wild-type 3′UTR reporter and negative control miRNA precursor (pre-miR-control). The results are obtained from triplicate experiments and presented as mean ± SE. (TIF) [file pone.0056647.s001.tif]

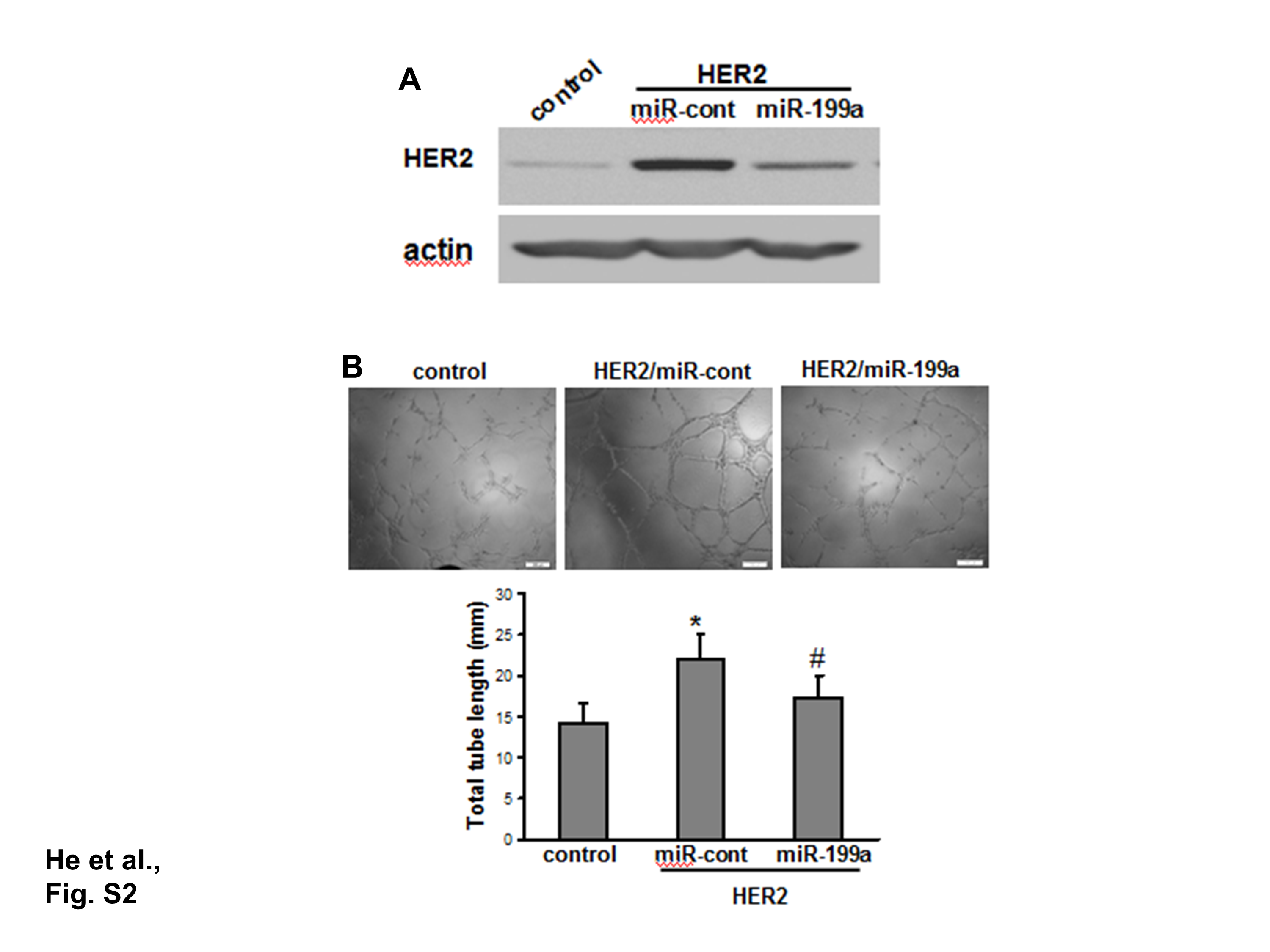

Supplement: Figure S2 — miR-199a inhibits tube formation in cells by overexpressing HER2 cDNA with 3′ UTR region containing miR-199a binding site. A) OVCAR-3 cells were infected with lentivirus carrying HER2 cDNA with 3′ UTR region, then transiently cotransfected with miR-control or miR-199a precursor as indicated. HER2 protein levels were determined 72 h after transfection by immunoblotting. B) HUVEC cells were cultured in serum free medium overnight and re-suspended in basic EBM-2 medium. The conditioned medium was prepared from OVCAR-3 cells as treated above. Tube formation assay was performed as described in Material and Methods. Upper: Representative images were shown. Scale bar: 200 µm. Lower: Total tube lengths (mm) were presented as mean ± SE from six replicates for each treatment. *Significantly different compared with control. #Significantly different compared with HER2/miR-cont. (TIF) [file pone.0056647.s002.tif]
